# Pre-Clinical Safety and Immunogenicity Study of a Coronavirus Protein-Based Subunit Vaccine for COVID-19

**DOI:** 10.3390/vaccines11121771

**Published:** 2023-11-28

**Authors:** Kamshat Shorayeva, Aziz Nakhanov, Ainur Nurpeisova, Olga Chervyakova, Kuanysh Jekebekov, Zhandos Abay, Nurika Assanzhanova, Sandugash Sadikaliyeva, Elina Kalimolda, Aibol Terebay, Sabina Moldagulova, Zharkinay Absatova, Ali Tulendibayev, Syrym Kopeyev, Gulnur Nakhanova, Aisha Issabek, Sergazy Nurabayev, Aslan Kerimbayev, Lespek Kutumbetov, Yergali Abduraimov, Markhabat Kassenov, Mukhit Orynbayev, Kunsulu Zakarya

**Affiliations:** Research Institute for Biological Safety Problems, The Ministry of Health of the Republic of Kazakhstan, Gvardeiskiy 080409, Kazakhstanabaizh097@mail.ru (Z.A.); elina.kalimolda@mail.ru (E.K.); zharkinay_a_s@mail.ru (Z.A.);

**Keywords:** COVID-19, subunit vaccine, antigens, toxicity, safety, immunizing dose, immunogenicity

## Abstract

Creating an effective and safe vaccine is critical to fighting the coronavirus infection successfully. Several types of COVID-19 vaccines exist, including inactivated, live attenuated, recombinant, synthetic peptide, virus-like particle-based, DNA and mRNA-based, and sub-unit vaccines containing purified immunogenic viral proteins. However, the scale and speed at which COVID-19 is spreading demonstrate a global public demand for an effective prophylaxis that must be supplied more. The developed products promise a bright future for SARS-CoV-2 prevention; however, evidence of safety and immunogenicity is mandatory before any vaccine can be produced. In this paper, we report on the results of our work examining the safety, toxicity, immunizing dose choice, and immunogenicity of QazCoVac-P, a Kazakhstan-made sub-unit vaccine for COVID-19. First, we looked into the product’s safety profile by assessing its pyrogenicity in vaccinated rabbit models and using the LAL (limulus amebocyte lysate) test. We examined the vaccine’s acute and sub-chronic toxicity on BALB/c mice and rats. The vaccine did not cause clinically significant toxicity-related changes or symptoms in our toxicity experiments. Finally, we performed a double immunization of mice, ferrets, Syrian hamsters, and rhesus macaques (Macaca mulatta). We used ELISA to measure antibody titers with the maximum mean geometric titer of antibodies in the animals’ blood sera totaling approximately 8 log2. The results of this and other studies warrant recommending the QazCoVac-P vaccine for clinical trials.

## 1. Introduction

The ongoing COVID-19 pandemic has created an urgent need to develop highly effective vaccines that would allow for controlling the public health situation, improve communities’ socio-economic statuses, and protect the population as a whole and high-risk groups, such as the elderly and medical staff, in particular [1,2,3,4,5,6]. 

Currently, several types of COVID-19 vaccine platforms are being tested. These include live attenuated vaccines; inactivated vaccines [7]; recombinant vaccines [8,9]; subunit vaccines [10]; synthetic peptide vaccines; and virus-like particle-, DNA-, and mRNA-based vaccines [11,12].

Genomic characterization research has established that SARS-CoV-2 has several open reading frames (ORFs) that encode accessory proteins, the non-structural replicase (ORF1a/ORF1b), as well as envelope (E), membrane (M), nucleocapsid (N), and spike (S) proteins [13,14]. Structural glycoprotein S expressed on the surface of the SARS-CoV-2 virus is a crucial factor in virus–host interactions and tissue tropism. These 180 kDa sized glycoproteins represent an important antigenic determinant capable of eliciting an immune response. As with other coronaviruses, the S-protein in SARS-CoV-2 facilitates host cell receptor recognition, cell attachment, and fusion during infection [15,16,17,18]. The S1 subunit contains a receptor-binding domain (RBD) that interacts with the ACE-2 receptor initiating a series of conformational changes that promote fusion and cell penetration [19]. The S2 subunit, which consists of FP, HR1, HR2, TMD, and consecutive cytoplasmic domains, plays a decisive role in the virus’s fusion and cell entry [14].

For this reason, subunit vaccines that have purified immunogenic viral proteins S1 and S2 of SARS-CoV-2 are considered safe and stable, while they also do not contain additional ballast proteins and nucleic acids that can lead to adverse events after vaccination [20]. Moreover, recombinant subunit vaccines for MERS and SARS use the RBD protein [21,22] and are protein antigens, which makes them much safer than nucleic acid-based products.

Subunit vaccines for SARS-CoV-2 may provide an effective alternative approach in the fight against COVID-19 [23]. However, although the RBD protein is an efficacious and safe antigen candidate, the immunogenicity produced by RBD monomers is insufficient [24]. Therefore, given the urgent need to build an effective means of specific prophylaxis for COVID-19, Kazakh scientists, for the first time in the country’s history, developed a technology for producing a subunit vaccine that uses recombinant proteins’ RBD, which is the SARS-CoV-2 S-protein’s receptor-binding domain, and N, the nuclear phosphoprotein expressed in *E. coli*.

New vaccines’ safety and efficacy profiles vary depending on the type of production technology, the adjuvant used, and even the route of administration [25,26]. This paper describes a preclinical study of the toxicity and safety of QazCoVac-P, a subunit of the COVID-19 vaccine in the form of a mixture of recombinant SARS-CoV-2 viral proteins, developed in Kazakhstan and manufactured by the Research Institute for Biological Safety Problems.

## 2. Materials and Methods

### 2.1. Ethics 

Our animal experiments complied with national and international laws and guidelines for handling laboratory animals. In addition, the study protocol was approved by the Committee on the Ethics of Animal Experiments of the Research Institute for Biological Safety Problems (Permit Number 1010/22).

### 2.2. Vaccine

This paper reports on a preclinical safety and immunogenicity study of QazCoVac-P, an intramuscular subunit vaccine for COVID-19 in the form of a mixture of recombinant RBD/N polypeptides (S-protein RBD and nuclear phosphoprotein of the SARS-CoV-2 coronavirus) that contains the AddaS03 adjuvant (InvivoGen, San Diego, CA, USA). The product is manufactured by the Research Institute for Biological Safety Problems (Table 1).

The AddaS03™ adjuvant (InvivoGen, San Diego, CA, USA) is an oil-in-water nanoemulsion that consists of two biodegradable oils: squalene and dl-α-tocopherol. Squalene emulsions of this type induce cellular (Th1) and humoral (Th2) immune responses. Substances included in the AddaS03 adjuvant (InvivoGen, San Diego, CA, USA) enable the use of doses with a low antigen concentration and are approved for human vaccines.

The buffer solution regulates the concentration of hydrogen ions (pH) in biological fluids and medicines.

### 2.3. Safety 

#### 2.3.1. Bacterial Endotoxin (BE) Content and Pyrogenicity Assessment 

Apyrogenicity is a crucial safety characteristic that has to be tested in all injectable products, including vaccines. The standard pyrogenicity assessing methods required by the European Pharmacopoeia are the rabbit pyrogen test (RPT), the bacterial endotoxin (BE) test (the LAL test), the recombinant factor C test, and the monocyte activation test (MAT) [27,28,29]. These methods are mandatory for all European countries. However, the State Pharmacopoeia of the Republic of Kazakhstan requires using only two pyrogenicity assessment methods—the LAL test and in vivo pyrogenicity experiments in rabbits [30,31].

In this preclinical study of QazCoVac-P’s safety, apyrogenicity and purity from BE, we used the vaccine’s bulk products made from recombinant polypeptides SARS-CoV-2 virus’s S-protein receptor-binding domain (VBP-RBD) and nuclear phosphoprotein (VBP-N) with no adjuvant, since AddaS03™ (InvivoGen, San Diego, CA, USA) is produced in strict aseptic conditions, it is sterile, and its endotoxin content does not exceed <10 EU/mL [32].

##### Pyrogenicity

We evaluated the pyrogenic properties of the tested product by measuring body temperature in rabbits as required by the State Pharmacopoeia of the Republic of Kazakhstan [31,33]. We experimented on 78 healthy mature rabbits of both sexes weighing 1.5–2.5 kg that we randomly assigned to groups of 3. 

We administered the tested product into the models’ marginal ear vein in the volume of 0.5 mL per 1 kg of body weight. A day before the start of the experiment, we measured the rabbits’ body temperatures and stopped feeding them while allowing them to access water. Before use, we heated a vaccine sample to 35 °C and introduced it within 15–30 min following the latest body temperature measurement. After administering the vaccine, we measured the animal’s body temperature every hour for 3 h. 

We considered a sample to be apyrogenic when the cumulative increase in the body temperature of all three rabbits in a group was less than or equal to 1.15 °C. Conversely, when it exceeded 1.15 °C, we considered it to contain pyrogens.

##### LAL-Test

We assessed the BE quantities in the vaccine utilizing the LAL test. We used the LAL reagent, endotoxin-free water, endotoxin-free tips, and endotoxin-free borosilicate glass tubes manufactured by Charles River (Charleston, SC, USA). The standard control endotoxin (CSE) we used was a lipopolysaccharide (LPS, endotoxin) from Escherichia coli strain O55:B5 obtained from Charles River Endosafe (Lot No. EX03492). The same company (Charles River EndosafeTM, Charleston, SC, USA) obtained the LAL reagent.

After incubating the tubes for 60 min at 37 °C, we inverted them 180° to check for a solid gel-like clot, representing a positive result. We defined the sensitivity of the clotting assay as the lowest CSE concentration (0.06 EU/mL) to produce a positive result.

#### 2.3.2. General Toxic Action 

To assess the general toxic action of the biological product, we chose the following tests: the local irritant action test, the acute toxicity test, and the sub-chronic toxicity test. We used a commercial PBS as a comparator. In these tests, we used outbred rats and mice that we assigned randomly to each experimental and control group.

##### Local Irritant Action 

We assessed the vaccine’s skin irritant action by conducting experiments on rats of both sexes weighing 200 ± 10.0 g. We randomly selected and divided laboratory animals into two groups (*n* = 10).

We shaved an area of approximately 5 cm^2^ around the injection site of each animal twenty-four hours before the experiment. In performing the procedure, we tried to avoid injuring the animals’ skin.

We administered the vaccine undiluted in the amount of 0.5 mL by injecting it at the site we had prepared. Thereafter, we observed the skin for 12 h.

On the next day after the procedure, we euthanized the models by exposing them to CO_2_. We took a 15 × 15 mm skin sample from the injection site of each animal. We washed the skin samples in saline and placed them in a 10% formalin solution for a histology examination.

##### Acute Toxicity 

We assessed the vaccine’s acute intramuscular toxicity by administering a single human dose in mice of both sexes after fasting them overnight (i.e., leaving them no access to food and letting them have only water for approximately 12–15 h). We then performed an acute intramuscular toxicity experiment on rats where we introduced 10 human doses into the animal models of both sexes. We divided the models randomly into two groups: an experimental and a control group. As a comparator substance, we administered PBS to the control group. We fed the models 4 h after administering the vaccine and the comparator substance. We observed the animals for clinical signs and death the first 2 h after the injection, then every 2 h until the end of day 1, and then on day 2, day 7, and day 14. We monitored body weight changes in the models on days 0, 2, 7, and 14. On day 14, we sacrificed all animals and performed a macroscopic observation of their organs and tissues. We fixed the organs in a 10% neutral buffered formalin and performed their histopathological examination [33].

##### Subchronic Toxicity 

**Figure 1:**
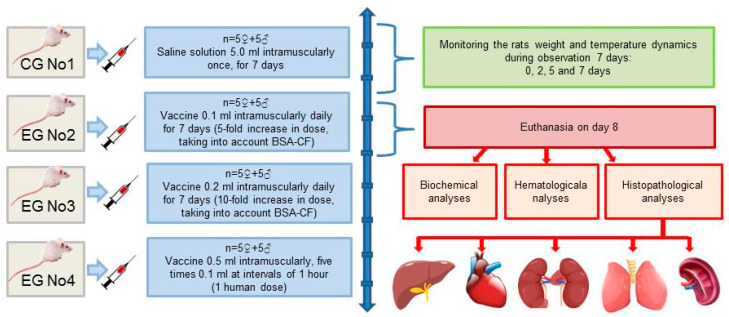
Subchronic toxicity test flowchart.

In this experiment, we used 40 randomly selected rats of both sexes weighing 180–200 g. We divided the animals into four groups (one control and three experimental groups, *n* = 10) and administered different vaccine doses intramuscularly in each experimental group, as shown in Figure 1. All the vaccine doses we used in this experiment had been selected in accordance with the RK GLP standard [1]. The introduction of the highest dose was expected to cause possible toxic effects or death in some of the animals.

To analyze the dynamics of the model animals’ body weight, we weighed them when assigning groups one day before the first injection and then on day 2 and day 7 of the experiment. We also monitored for alterations in the animals’ behavior after vaccination, and then looked for histopathological and morphological changes in their organs, such as the heart, lungs, liver, spleen, and kidneys, after necropsy.

### 2.4. Safety Testing, Assessing the Immunizing Dose and Immunogenicity 

**Animals**

In our work to evaluate the vaccine’s safety, determine its immunizing dose, and assess its immunogenicity, we used macaques (Macaca mulatta) aged 6–8 years (*n* = 3). We performed the vaccine’s immunogenicity assessment on healthy 6–8-week-old Syrian hamsters of both sexes (*n* = 40) and 6–7-month-old male ferrets (*n* = 6). All of these model animals were obtained from special breeding facilities in Russia and Kazakhstan. The models were randomly assigned to groups. As our criteria for the acceptability of randomization, we used the absence of external signs of disease and the groups’ body weight homogeneity (±20%).

**Safety testing, determining the immunizing dose and assessing the vaccine’s immunogenicity in healthy monkeys (Macaca mulatta)**

In this experiment, we administered QazCoVac-P to healthy macaques intramuscularly twice, 21 days apart. We tested the vaccine in three doses (2.5 µg, 5.0 µg, and 10.0 µg RBD-N/animal in a volume of 0.5 mL). We had as our control models 3 non-immunized monkeys of the same age as the experimental animals. We handled the animals per the European Convention for the Protection of Vertebrate Animals used for Experimental and Other Scientific Purposes and domestic regulations.

Before immunizing the models, we anaesthetized them with an intramuscular injection of 0.03–0.04 mL of Zoletil (Virbac, Carros, France) at a concentration of 100 mg/mL.

In the experiment, we observed both vaccinated and control group animals (monitoring each animal’s body temperature, weight, and general condition) for 21 days after each immunization. Also, on day 14 and day 21 after the first immunization and on day 7 after re-immunization, we collected the animals’ blood samples to test them for virus-specific antibodies using the ELISA.

**Assessment of immunogenicity in laboratory models (Syrian hamsters, ferrets)**

We performed an assessment of the vaccine’s immunogenicity by administering it in two intramuscular injections of 0.5 mL 21 days apart. As a comparator substance, we used PBS. Both the vaccinated and control group animals underwent clinical observation as part of the experiment. On days 14 and 21 after the first immunization and on days 7 after re-immunization, we collected the animals’ blood samples to test them for specific antibodies using the ELISA.

**Assessment of Immunogenicity in ELISA**

We used the ELISA test to assess the titer of SARS-CoV-2 antibodies in serum samples from immunized animals. To set up ELISA, we sensitized 96-well plates with recombinant proteins at 2 µg/mL overnight at 4 °C. We washed the plates 3 times with 200 µL of TBST buffer (150 mM NaCl, 20 mM Tris-HCl, pH 7.5, 0.05% Tween 20) (Sigma-Aldrich GmbH, Taufkirchen, Germany). For blocking, we added 200 µL of BB buffer (1% BSA per TBST) into each well and incubated the plate for 1 h at room temperature. We diluted test sera with a 1:50 BB buffer and added 100 µL per well. We incubated the plates for 2 h at room temperature, then washed them 4 times with 200 µL of TBST buffer. This was followed by adding secondary antibodies labelled with alkaline phosphatase at the manufacturer-recommended dilution and a 1 h incubation at room temperature. We washed the plates 4 times with 200 µL of TBST buffer and added 100 μL of pNPP substrate. Then, we incubated them in dark conditions for 1 h. We read the results 20 to 30 min after adding the substrate using an ELISA analyzer at a wavelength of 405 nm.

### 2.5. Statistical Analysis

We analyzed all experimental data statistically using Graph Pad Prizm Software version 8.0 (Graph Pad Software Inc., La Jolla, CA, USA). We determined the experiments to be repeatable and the results to be reproducible.

We compared the differences in protective efficacy between groups using one-tailed Fisher’s exact test, with *p* < 0.05 considered significant, and mean values presented with standard errors (SEM). 

We assessed the vaccine’s immunogenicity in the different groups by determining the antibody geometric mean titer (GMT) at a confidence interval of 95%. The mean values in this paper are reported with standard errors (SEM). We determined the significance of the difference between groups by using a two-way ANOVA, with significance expressed as * *p* < 0.05. We used a one-way analysis of variance (Dunnett’s test) to compare dose-dependent changes and body weight dynamics in the experimental groups.

## 3. Results

### 3.1. Safety

An important criterion used in determining whether an injectable immunobiological preparation is safe or not is its apyrogenicity. The apyrogenicity of injectable products is characterized by their purity from BE of Gram-negative bacteria that cause an increase in body temperature in models such as rabbits when administered intravenously.

#### 3.1.1. Assessing BE Quantities and Pyrogenicity 

**Figure 2:**
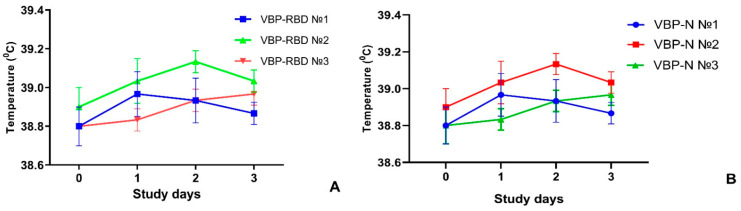
Body temperature of rabbits after intravenous administration of VBP-RBD (**A**) and VBP-N (**B**) in the study of the QazCoVac-P bulk-product pyrogenicity.

We performed the pyrogenicity assessment of experimental batches of QazCoVac-P in accordance with the State Pharmacopoeia of the Republic of Kazakhstan; the results of this assessment are presented in Figure 2.

As shown in Figure 2 above, the difference between the maximum and background body temperatures in rabbits immunized with recombinant RBD protein-containing QazCoVac-P bulk product was at 0.1–0.3 °C, while the total increase in body temperature with this particular product was 0.5–0.7 °C. With the recombinant N protein-based QazCoVac-P bulk product, the difference between the maximum and the background body temperatures was at 0.1–0.2 °C, while the total increase equaled 0.5–0.6 °C. Thus, we ascertained that the tested samples of the QazCoVac-P vaccine’s bulk products containing recombinant proteins were apyrogenic. 

In the subsequent series of experiments, we confirmed bulk product samples’ apyrogenicity by assessing BE quantities using the LAL test. The results are presented in Table 2.

The BE quantity assessment results in Table 1 demonstrate that the tested bulk product samples did not differ in their bacterial endotoxin quantities, did not exceed the permissible level (less than 110 IU/mL), and met RK SP standards.

#### 3.1.2. General Toxic Action 

##### Local Irritant Action Assessment 

We noted no changes at the animals’ injection site or any pronounced pathological changes such as hyperemia, swelling, erythema, peeling, infiltration, irritation, or tissue necrosis after administering the vaccine. The animals’ skin at the injection site was normal, with no signs of irritation. At the end of the experiment, we decapitated all animals to assess the macro- and microscopic skin changes. This examination found that the skin and subcutaneous tissue at the injection site were unchanged. There were no hemorrhages, infiltration, irritation, or tissue necroses observed. 

**Figure 3:**
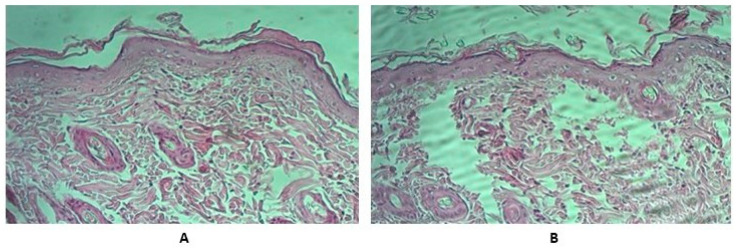
Typical results of samples’ pathological examination (HE × 200). Hematoxylin and eosin staining. (**A**) Control group skin sample. (**B**) Experimental group skin sample. We shaved an area of approximately 5 cm^2^ around the injection site of each animal 24 h before the experiment. We tried to avoid injuring the animals’ skin in the procedure. We administered the vaccine undiluted in the amount of 0.5 mL by injecting it at the site we had prepared. Thereafter, we observed the skin for 12 h.

We performed a pathomorphological examination of rats’ skin and tissues adjacent to the injection site. We fixed the collected skin samples in a 10% formalin solution and stained ready sections with hematoxylin and eosin. Microscopy of skin samples from control group animals showed that the skin preserved its histological structure and had only small dermal edema foci (Figure 3).

##### Acute Toxicity 

As we performed the acute toxicity assessment, we observed no deaths in animals, including those given maximum allowable doses, from the beginning to the end of the experiment. The results are presented in Table 3.

**Figure 4:**
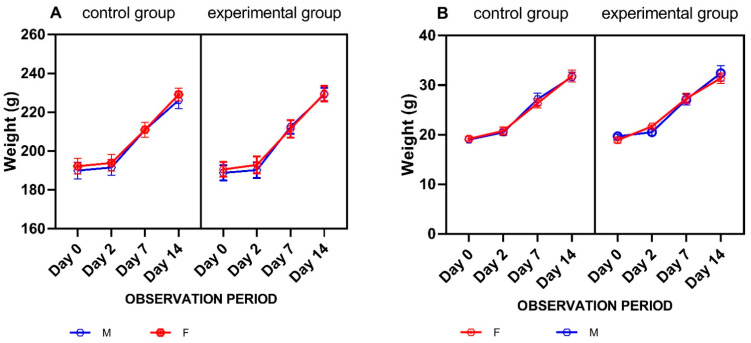
Body weight dynamics in rats (**A**) and mice (**B**) immunized with the vaccine candidate. Each value represents the mean ± SEM of all animals in each group.

Throughout the experiment, we observed no changes in the live weight of model animals. The body weight dynamics in the experimental group did not differ from that of the control group. All animals demonstrated an increase in body weight, as can be seen in Figure 4.

We detected no deviations from normal vital signs in any experimental animal on the first or subsequent days of observation. The animals appeared healthy and responded appropriately to tactile, auditory, and light stimuli; their fur was shiny, even, and smooth. 

In this experiment, we found that mice and rats of both sexes tolerated the maximum allowable volume of the vaccine tested (rats—5.0 mL, mice—0.5 mL) without any visible deviations from the norm.

##### Subchronic Toxicity and Necropsy Data 

At the end of the subchronic toxicity experiment, the general condition of animals in the experimental group was not visually different from that of the control group animals. The intensity and nature of the motor activity, the motor coordination, and the skeletal muscle tone of the experimental group animals remained at the same level throughout the experiment. Their behavioral reactions, as well as their response to tactile, pain, auditory, and light stimuli, were unchanged. The hair and skin were in normal condition. The results of our subchronic toxicity experiment and data on the vaccine’s impact on the survival of laboratory rats are presented in Table 4.

Throughout the experiment, we weighed the animals as per the study protocol. The dynamics of the experimental group animals’ body weight are presented in Table 5 below. The data demonstrate an increase in the body weight of the experimental group animals during the entire observation period. We found no statistically significant differences in the experimental group animals’ body weight compared to the control group.

The values are expressed as mean ± SEM (=10 for each group). When using the one-way analysis of variance followed by Tukey’s multiple comparison tests, we considered * *p* values < 0.05 as significant.

We euthanized the animals of all groups by exposing them to CO_2_ fifteen days after the end of the experiment. A macroscopic examination of the internal organs showed no differences between the groups. The animals’ fur was neat and shiny and had no bald areas. Examining the chest and abdominal cavities identified no anomalies in their internal organ location. The two groups had no difference in the appearance of animals’ internal organs. The size and shape of the heart in the experimental group animals were unchanged. The heart muscle was brownish and dense. The lungs were of a pale-pink color and the tissue in the section area was also of a uniform pale-pink color, while the lungs’ surface was smooth.

The stomach had a normal shape and size; its lumen was filled with dense food contents. The mucosa of the stomach was pale pink, shiny, and folded. 

The size or shape of the liver was unchanged. The liver tissue was of a brownish color and a moderately firm texture. The pancreas was pale pink and lobed.

The size and shape of the kidneys in experimental group animals did not differ from those of the control group, and their capsule could be removed easily. The surface of the kidneys was smooth and had a uniform brownish-grey color. The shape, size, and density of the adrenal glands, ovaries, or testicles were not different from the norm.

The spleen had a dark cherry color, smooth surface, and dense texture. The membranes of the brain were thin and transparent. The brain matter was moderately dense. 

A histological examination performed as part of the subchronic toxicity experiment showed that the introduction of toxic doses (0.1 mL, 0.2 mL and 0.5 mL) had led to microscopic changes in organ tissues in the form of minor circulation disturbances such as vascular hyperaemia and tissue edema. We found no evident pathological changes in any of the organs examined. 

We performed statistical data processing on the relative weight of internal organs of animals in the experimental groups, comparing them with that of the control group (Table 6). 

As seen from Table 6, there were no significant differences in the weight coefficients of the experimental group animals’ internal organs, which indicates the absence of any toxic effect on the animals’ bodies from the tested vaccine.

We collected blood samples for biochemical and morphological analyses on the eighth day of the subacute toxicity experiment. The results of these are presented in Table 7 and Table 8. Also, we observed no changes at the injection site immediately after vaccine introduction. 

As seen in Table 7, the biochemical parameters of blood samples from all experimental group animals fall within the normal range, except for alkaline phosphatase for Group 4. In Group 4, this parameter was three times higher than in the control group. This could be due to the fact that animals in this group received five 0.5 mL doses of the vaccine within one day, and it produced its toxic effect on the liver and the biliary tract. However, we saw no deaths in this group. In Group 3, we noted a decrease in the total bilirubin level, which was associated with the occurrence of a pathological process in the body, but this difference in the total bilirubin level was not significant. Glucose levels in all of the experimental groups were virtually the same as in the control group.

An analysis of the morphological composition of experimental rats’ blood showed that all hematology parameters of the control and experimental Group 2 were within the physiological norm (Table 8). However, we observed an increase in the levels of granulocytes and platelets in Group 3, as well as a slight upturn in the large platelet (P-LCC) level and erythrocyte sedimentation rate (ESR) in Group 4. These deviations may be due to the toxic effect of the multiple immunizations and the animals’ response to them. It is quite possible that these parameters can subsequently normalize. However, ascertaining the vaccine’s toxic action on the internal organs and systems will require a separate study assessing its individual effects on a living organism. This should be taken into account when conducting clinical trials. The rest of the blood morphology parameters in all of the groups were within physiological norms.

Even though the experimental animals were repeatedly exposed to the vaccine as we administered it every day for 7 days, we did not observe any change in their general state, orientation and motor activity, or their emotional status, as evidenced by a positive change in the body weight of both control and experimental group animals throughout the experiment. Also, the subacute toxicity experiment resulted in no pathological alterations in the liver, kidneys, heart, spleen, and lungs, as proven by the histology. A pathomorphological examination of the animals’ internal organs showed no signs of inflammation or tissue destruction at the injection site, nor did it reveal any macroscopic/microscopic alterations or hypervolemic edema in the internal organs, as confirmed by the values of their weight coefficients. However, the hematological and biochemical tests showed changes in several blood parameters (GRA, PLT, P-LCC, and ESR). Although we observed no visible deviations in the animals’ behavior, the slight changes above indicate the animals’ immune response to the vaccine.

### 3.2. Safety Testing, Assessing the Immunizing Dose and Immunogenicity

#### 3.2.1. Assessing the Immunizing Dose 

In the 14 days of monitoring the vaccine’s safety in rhesus monkeys (Macaca mulatta), after a single-dose injection, no animal in any of the groups had any somatic or neurological deviations, altered their food and/or water consumption patterns or died. None of the vaccinated animals developed any clinical or neurological symptoms; and the weight of each animal on the final day of observation was no lower than in the control group.

**Figure 5:**
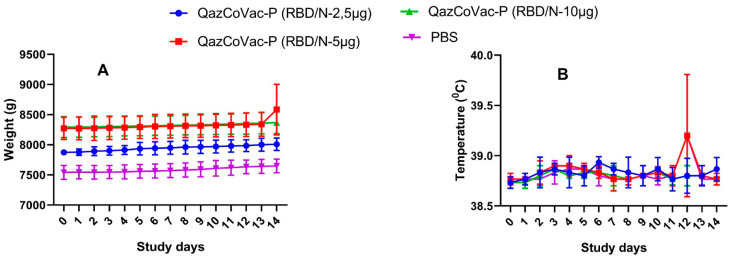
Live weight and body temperature parameters of rhesus monkeys after double immunization. The observation period lasted 14 days; the standard deviations (SD) for the groups’ mean body temperature values are provided as error bars. (**A**) Body weight dynamics of rhesus monkeys. (**B**) Dynamics of changes in body temperature.

The dynamics of animals’ body weight did not differ significantly between the experimental and control groups (Figure 5). The rate of body weight growth in the experimental group animals after a single vaccination was comparable with that of the control group. Also, the body weight growth rate in each individual group remained unchanged from day 1 to day 14 of observation. These data are of significant interest since the tested vaccine does not produce a negative effect on the rhesus monkeys’ body weight.

The body temperatures of rhesus monkeys during 14 days after the first vaccination were normal in both the experimental and control groups, with no particular fluctuations (Figure 5). In summary, experiments on rhesus monkeys demonstrated that the QazCoVac-P vaccine was safe at all doses tested. 

**Figure 6:**
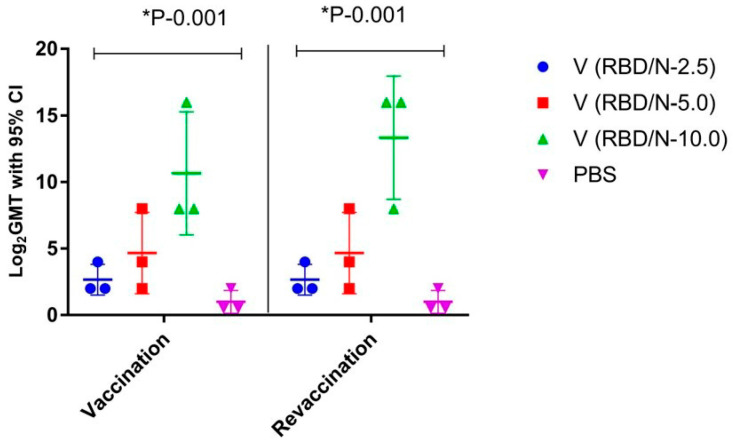
Virus-specific antibody titers in the blood sera of rhesus monkeys immunized with different doses of the antigen.

We went on to assess the vaccine’s immunizing dose in rhesus monkeys. We used the following antigen doses for the different groups: 2.5 µg, 5.0 µg, and 10.0 µg per animal, all in a volume of 0.5 mL. The experiment resulted in significant differences between the control and experimental groups, as well as an increase in the antibodies’ geometric mean titer (Figure 6). When analyzing the GMT in Dunnett’s multiple comparisons test at a 95% confidence interval following the first immunization, we observed statistically significant differences between the groups—the 2.5 μg group, the 5.0 μg group, the 10.0 μg group, and the control group vaccinated with PBS (*p* = 0.001).

Thus, a single and double administration of the vaccine with various antigenic loads contributed to an increase in the antibody titer, with an insignificant variance of GMT between groups immunized with different quantities of the antigen. 

**Figure 7:**
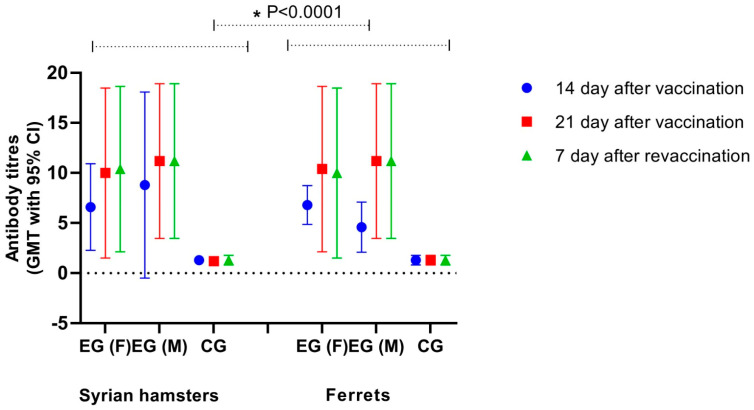
Antibody response in vaccinated Syrian hamsters and ferrets. GMT of SARS-CoV-2 antibodies as assessed by ELISA. The analysis of GMT using Dunnett’s multiple comparisons test with a confidence interval of 95% after the first and second injection identified statistically significant differences between the experimental group and the control group, in which PBS was administered instead of the vaccine (* = *p* < 0.0001). The bars show an increase in antibodies on days of observation in both gender groups. On day 14 and 21 after the first vaccination and on day 7 after re-vaccination, no specific antibodies to PBS were detected.

The above examination of the vaccine’s immunogenic properties showed that regardless of the amount of antigen contained in the doses, a double immunization of monkeys with QazCoVac-P through the intramuscular route leads to them developing an immune response (Figure 7). In the negative control group, where animals received PBS instead of the vaccine, we detected no antibodies with ELISA, or if we did, their value was less than 1:2. A dose of 10.0 µg/individual formed antibodies with a titer of more than 8 log2 in all macaques in the group.

#### 3.2.2. Examining the Vaccine’s Immunogenicity in Syrian Hamsters and Ferrets

We also examined QazCoVac-P’s immunogenicity in Syrian hamsters and ferrets as we administered two 0.5 mL doses intramuscularly 21 days apart. We collected the animals’ blood sera for immunogenicity examination with ELISA on day 14 and day 21 after the first vaccination and on day 7 after re-vaccination (Figure 7).

We observed statistically significant differences between experimental groups (Figure 7). Moreover, the most pronounced immune response to the QazCoVac-P vaccine could be seen in all groups 21 days after the first vaccination.

## 4. Discussion

Following the WHO’s recommendations, many countries developed their vaccine candidates to combat COVID-19 as the pandemic took hold. These have included conventional inactivated vaccines, live attenuated vaccines, recombinant vaccines, synthetic peptide vaccines, virus-like particle-based vaccines, DNA and mRNA-based vaccines, and subunit vaccines containing purified SARS-CoV-2 S1 and/or S2 immunogenic viral proteins. The latter are considered to be some of the safest and most stable products free from additional ballast proteins and nucleic acids capable of causing adverse reactions. In their study of a candidate RBD homodimeric vaccine, Chinese researchers found that a high titer of antibodies in rodents and rhesus monkeys developed after three vaccinations. In this study, mice developed specific antibodies after the second vaccination (on day 28) [34].

A decisive role in the fight against infectious diseases is given to specific preventions using various vaccines. Research on creating new-generation vaccines against the coronavirus infection COVID-19 is critically important for the healthcare system of the Republic of Kazakhstan. Multiple vaccines are being developed around the world to this day. However, before mass vaccination can begin, all vaccines must be shown to be safe and effective in large-scale clinical trials. The latest achievement in molecular biology, genetic engineering, and biotechnology has been the creation of vaccines containing highly immunogenic viral proteins. Vaccines based on recombinant proteins are the newest generation of preventive drugs. The development of vaccinology makes it possible to use new technologies in the development of vaccines. This is a recombinant subunit vaccine technology. Vaccine development research began with the selection of immunogenic viral proteins.

Several viral antigens are used to create experimental vaccines. The focus is on the surface protein S because of its vital role in infectious activity. In addition, nucleocapsid protein N, envelope protein E, or nonstructural protein 16 (NSP16) are used as a source of antigens. As a result of the analysis of the literature data, two components were selected—the RBD fragment of the receptor binding domain of the protective antigen S protein and the nucleophosphoprotein N protein of the SARS-CoV-2 coronavirus, which is responsible for the cellular immune response [35,36].

Subunit vaccine candidates in development are mainly based on the Spike protein or the receptor-binding domain (RBD) from SARS-CoV-2. The RBD is located within the S1 subunit of the Spike. Angiotensin-converting enzyme 2 (ACE2) is the functional receptor for SARS-CoV-2 comprising a critical factor for SARS-CoV-2 to enter into target cells, and RBD is a key functional component that is responsible for the binding of SARS-CoV-2 to host cells. It is therefore not surprising that antibodies directed against the RBD or overlapping with the ACE2 binding region are strongly neutralizing, making the RBD a promising antigen (Ag) for subunit vaccines (10). RBD-based antigens have been described in previous studies for the SARS-CoV and MERS-CoV vaccine development. The RBD from SARS-CoV-2 is an ideal Ag for vaccine formulations because of its high expression levels, ease of manufacturing, stability, and capacity to elicit functional antibodies [37].

The target genes of the SARS-CoV-2 coronavirus were amplified and cloned into pET vectors for bacterial expression. *E. coli* ER2566 cells were transformed with the constructed recombinant plasmids pRBD and pN. As a result of the induction of bacterial cells containing recombinant plasmids pRBD and pN, the RBD and N proteins with molecular masses of 23 and 46 kDa, respectively, were obtained. A Western blot analysis of cell lysates using sera obtained from people who had recovered from coronavirus infection confirmed the specificity of the recombinant proteins.

Protocols for the expression and purification of recombinant proteins were developed and optimized to obtain preparation quantities of recombinant proteins. To enhance the immune response, the AddaS03 nanoemulsion adjuvant approved for humans was included in the vaccine. Here, we demonstrated that the addition of AddaS03 nanoemulsion to the RBD and N proteins formulation was able to increase the induction and increase in SARS-CoV-2 virus-specific antibody responses. Safety and stability studies will be necessary to evaluate the formulation’s safety for human use. Our results suggest that adding ADD033 could enhance vaccine formulations containing an antigen and adjuvant [38,39,40,41,42].

The WHO has issued recommendations that each country should develop its own vaccines and expand their vaccine production capacities. Over the past several years, the Scientific Research Institute for Biological Safety Problems (RIBSP) has been doing re-search to develop vaccines against influenza and *M. tuberculosis* for the Ministry of Health of the Republic of Kazakhstan [43,44,45,46]. Like many other countries, Kazakhstan started designing its own SARS-CoV-2 vaccine immediately after the COVID-19 outbreak with several candidate vaccines proposed by the RIBSP [47,48,49,50]. One of these was a subunit vaccine called QazCoVac-P, which is being examined here. In terms of its quality, QazCoVac-P is not inferior to vaccines developed in other countries.

Experimental batches of the vaccine were prepared to study the safety and immunogenicity of the subunit vaccine. Studies were also conducted to determine the stability of the nanoemulsion and the preservation of vaccine samples under various temperature and time storage conditions. The nanoemulsion remained stable at 37 °C throughout the entire observation period (14 days), and no separation into fractions was observed during centrifugation.

The subunit vaccine was harmless to laboratory animals and produced specific antibodies in the animals’ bodies. The vaccine remained stable for 3 months (observation period) at 4 °C.

Animals have always been an important investigation tool in medical research. The use of animals enables researchers to carry out safety, toxicity and immunogenicity, and other testing of vaccines. This article describes the results of our preclinical safety, immunogenicity, and dosage study of QazCoVac-P, a subunit vaccine for COVID-19. To achieve the goal of our study, we used rabbits, mice, rats, Syrian hamsters, ferrets, and rhesus monkeys (Macaca mulatta), as required by applicable regulations [50].

COVID-19 vaccines’ quality is safeguarded by existing national authorities’ rules based on WHO requirements. These rules are concerned with the specific activity, residual cell DNA, sterility, safety and, in particular, bacterial endotoxin (BE) count and pyrogenicity. The apyrogenicity of an injectable immunobiological preparation is expressed as purity from bacterial endotoxins which cause an increase in the body temperature of rabbits that received the vaccine intravenously. Bacterial endotoxins are toxic substances in Gram-negative bacteria’s external membrane which are released during lysis (decomposition) of the bacterial cell and pose a threat for the individual’s health by causing a series of immune responses. Being a potent immune stimulator, endotoxins can activate a number of mammal immune cells, especially once in the circulation system, and cause the over-secretion of a wide range of cytokines. This may cause multi-organ insufficiency, i.e., a severe non-specific stress response of the body, septic shock, or even death. BE contamination is a serious issue that affects hundreds of thousands of people annually throughout the world. Also, BEs are stable and can withstand extreme pH, high temperature, and even autoclaving. Thus, quantifying and controlling BE, which is one of the objectives of this study, is an important criterion used in the mass production and quality control of medications and immunobiological preparations [51,52,53]. 

Safety of use is the main requirement for new vaccines; this includes pyrogen count as a key quality and safety characteristic that should be tested in all injectable products, including vaccines. According to the European Pharmacopoeia, standard pyrogen testing methods include the rabbit pyrogen test (RPT), the LAL test, the recombinant factor C test, and the monocyte activation test (MAT) [26,27]. These methods are seen as canonical by all countries and their regulations. Even though there are differences in requirements for animals [54,55,56,57], experiment protocols, and LAL tests’ positive result interpretation [58,59,60,61], the goals of these methods are the same in all countries. The advantages of the LAL test include a high sensitivity, simplicity of use, reliability, good reproducibility, and a high throughput within a short time. When used en masse in large-scale operations, this method is undoubtedly cheaper than the rabbit test. 

The State Pharmacopoeia of the Republic of Kazakhstan provides for the possibility of using alternative pyrogen testing methods, such as an in vitro bacterial endotoxin test using LAL reagent produced from horseshoe crabs (*Limulus polyphemus*), as well as in vivo assessment of pyrogenicity in rabbits [29].

We performed the pyrogenicity assessment of experimental batches of QazCoVac-P in accordance with the State Pharmacopoeia of the Republic of Kazakhstan. The pyrogenic properties of the product was tested by measuring the body temperature in rabbits. In this preclinical study of QazCoVac-P’s safety, apyrogenicity, and purity from BE, we used the vaccine’s bulk products made from recombinant polypeptides SARS-CoV-2 virus’s S-protein receptor binding domain (VBP-RBD) and nuclear phosphoprotein (VBP-N) with no adjuvant, since AddaS03™ is produced in strict aseptic conditions, it is sterile, and its endotoxin content does not exceed <10 EU/mL. 

Thus, we found that the tested experimental series of the vaccine’s bulk products were nonpyrogenic in rabbits, as the National Pharmacopoeia of the Republic of Kazakhstan defines a medicine as apyrogenic if the sum of three vaccinated rabbits’ body temperature does not exceed 1.15 °C. The BE quantity assessment indicates that the endotoxin count of ≥3 EU/mL that we detected was significantly lower than the maximum allowable endotoxin concentration of 100 EU/mL as provided by the State Pharmacopoeia of the Republic of Kazakhstan for the studied preparation. This degree of purity from endotoxins allows for the intramuscular use of the preparation.

Sub-chronic toxicity studies usually take 9 to 12 months because some substances can manifest their toxicity only after several exposures to it. According to WHO guidelines, the duration of studies in animals should be based on the expected period of clinical use of the tested substance in people. The study assesses the effect on the target organs. The results of the study can be used later as confirming data in planning further clinical studies [62].

We performed an assessment of QazCoVac-P’s local irritating effect using white outbred rats of both sexes and detected no damaging processes except some focal dermal edema.

In the acute toxicity experiment, we gave mice and rats a single vaccine injection in 1 and 10 doses per animal, respectively, and observed no deaths from toxicity in either experimental group. The animals had no external signs of intoxication and did not lose weight across the entire observation period (14 days).

It is a known fact that the body weight is a sensitive indicator of xenobiotics’ toxicity in a wide range of toxicology studies [63]. In this study, the vaccine did not affect the increase in the body weight of animals in the experimental groups except the sixth experimental group.

A histological examination performed as part of the subchronic toxicity assessment found that introducing toxic doses of the vaccine (0.1 mL, 0.2 mL and 0.5 mL) led to microscopic changes in organ tissues characterized by minor circulation disturbances in the form of vascular hyperemia and tissue edema. We identified no evident pathological changes in any of the organs examined. 

According to the literature, the hamster is a widely used experimental model for many viral infections and has previously been reported to support the replication of the SARS-CoV virus [64,65,66].

The white mice, Syrian hamsters, ferrets, and rhesus macaques (Macaca mulatta) developed an immunogenicity profile on day 14 after the first immunization, a complete immune response after the second immunization, and a further increase in the immune response on the 28th day of the study as assessed by the MNA and ELISA tests. Similar results were seen in other inactivated candidate vaccines for SARS-CoV-2. Macaques immunized with the candidate PiCoVacc vaccine, for example, displayed a partial or complete immune response between the first and third PiCoVacc immunizations [5].

Our assessment of immune responses in animals vaccinated twice showed that the highest GMT of antibodies in their blood sera was equal to 8 log2.

In a similar study, researchers immunized six female Chinese rhesus monkeys aged 2–3 years with two doses of a dimeric RBD vaccine called PS-RBD (25 μg/dose) to assess its immunogenicity. The peak anti-spike antibody GMT after the first dose was 42.847. Anti-spike IgG significantly increased after the second dose of PS-RBD, and the GMT peaked on day 28 [67].

There have also been immunogenicity studies of a protein subunit vaccine called Nanocovax. In these studies, groups of BALB/c mice and hamsters received two doses (25, 50, 75, and 100 µg) of the vaccine containing 0.5 mg of Al3+ (aluminum hydroxide adjuvant) 7 days apart. In antibody tests, the titers of SARS-CoV-2 neutralizing antibodies in BALB/c mice and hamster models measured at 80–640 and 20–320, respectively [68].

In a morphological analysis of the blood of animals that we performed as part of our subchronic toxicity assessment, we found some differences in the composition of the peripheral blood of experimental group animals (GRA, PLT, P-LCC, and ESR) compared with the control group.

Based on the above work, we established that double immunization with the subunit vaccine yields the most effective results, can produce a high antibody titer in vivo, and thereby elicits a specific immune response to the coronavirus infection. 

The results of our study demonstrated that the QazCoVac-P vaccine was safe and produced a specific immune response to the SARS-CoV-2 in animal models such as Syrian hamsters and ferrets. The results of this and other studies provide grounds for recommending QazCoVac-P for clinical trials in volunteers.

## Figures and Tables

**Table 1:** Ingredients contained in one dose (0.5 mL) of the product.

Active Ingredient	Unit of Measure	Quantity
Spike protein receptor-binding domain	µg	10.0
Nuclear phosphoprotein	µg	10.0
**Accessory ingredients**		
AddaS03™ adjuvant	mL	0.25
Phosphate-buffered solution	mL	up to 0.5

**Table 2:** LAL test results.

#	Test Samples	Dilution		Standard Endotoxin Control Sample	Negative Control	Results
		Number of Dilutions	Endotoxin Count			Bulk Product
1	VBP based on recombinant N protein (SARS-CoV-2 nuclear phosphoprotein)	10^−1^	≥0.3 EU/mL	+ +	− −	+ +
2		10^−2^	≥3 EU/mL	+ +	− −	+ +
3		10^−3^	≥30 EU/mL	+ +	− −	+ +
4		10^−4^	≥300 EU/mL	+ +	− −	− −
5		10^−5^	≥3000 EU/mL	+ +	− −	− −
1	VBP based on recombinant RBD protein (SARS-CoV-2 spike protein receptor-binding domain)	10^−1^	≥0.3 EU/mL	+ +	− −	+ +
2		10^−2^	≥3 EU/mL	+ +	− −	+ +
3		10^−3^	≥30 EU/mL	+ +	− −	− −
4		10^−4^	≥300 EU/mL	+ +	− −	− −
5		10^−5^	≥3000 EU/mL	+ +	− −	− −
1	VBP based on recombinant proteins (RBD + N)	10^−1^	≥0.3 EU/mL	+ +	− −	+ +
2		10^−2^	≥3 EU/mL	+ +	− −	+ +
3		10^−3^	≥30 EU/mL	+ +	− −	+ +
4		10^−4^	≥300 EU/mL	+ +	− −	− −
5		10^−5^	≥3000 EU/mL	+ +	− −	− −

**Table 3:** Survival of mice and rats in acute toxicity experiment.

Groups	Name of Tested Substance, Volume, Frequency, and Route of Administration	Number of Dead/Total Number of Animals	Mortality (%)
Rats			
#1 Control group	PBS/5.0 mL, single, intramuscular	0/20	0.0
#2 Experimental group	Vaccine/5.0 mL, single, intramuscular	0/20	0.0
Mice			
#1 Control group	PBS/0.5 mL, single, intramuscular	0/20	0.0
#2 Experimental group	Vaccine/0.5 mL, single, intramuscular	0/20	0.0

**Table 4:** Animal survival in sub-chronic toxicity test experiment.

Groups	Number of Dead Animals/Total Number of Animals	Mortality (%)	Groups	Number of Dead Animals/Total Number of Animals	Mortality (%)
CG No1	0/10	0.0	EG No3	0/10	0.0
EG No2	0/10	0.0	EG No4	0/10	0.0

Notes: CG—control group; EG—experimental group.

**Table 5:** The dynamics of the experimental group animals’ (rats) body weight.

Group	Evolution of Body Weight (g)							
	D 0		D 2		D 5		D 7	
	♀	♂	♀	♂	♀	♂	♀	♂
CG No. 1	193.5 ± 2.5	192.0 ± 1.7	195.7 ± 2.4	194.1 ± 1.6	216.2 ± 1.0	214.78 ± 2.50	235.7 ± 0.92	230.86 ± 1.40
EG No. 2	186.76 ± 3.91	189.56 ± 3.95	188.66 ± 4.07	191.82 ± 3.46	216.24 ± 0.55	215.3 ± 1.92	232.54 ± 2.52	231.46 ± 1.07
EG No. 3	184.58 ± 1.4	194.34 ± 1.6	187.16 ± 1.39	196.58 ± 1.6	215.4 ± 1.84	216.06 ± 1.64	231.26 ± 0.39	232.02 ± 1.38
EG No. 4	184.38 ± 1.82	190.8 ± 3.36	187.18 ± 1.98	193.38 ± 3.42	215.76 ± 1.72	215.26 ± 2.5	235.28 ± 1.13	234.44 ± 0.63

Notes: CG—control group; EG—experimental group.

**Table 6:** Organ weights in rats.

Groups	CG No1	EG No2	EG No3	EG No4
Group size	*n* = 10	*n* = 10	*n* = 10	*n* = 10
Heart				
Mean weight ± S.D. (g)	1.19 ± 0.13	1.65 ± 0.16	1.19 ± 0.21	1.47 ± 0.12
Mean % body ± S.D. (g)	0.50	0.70	0.51	0.62
Liver				
Mean weight ± S.D. (g)	14.35 ± 3.97	13.75 ± 0.70	13.79 ± 0.89	15.59 ± 0.37
Mean % body ± S.D. (g)	6.08	5.91	5.96	6.62
Spleen				
Mean weight ± S.D. (g)	1.65 ± 0.52	2.17 ± 0.18	1.59 ± 0.21	2.13 ± 0.17
Mean % body ± S.D. (g)	0.70	0.93	0.68	0.90
Lungs				
Mean weight ± S.D. (g)	2.48 ± 0.53	2.25 ± 0.21	2.25 ± 0.37	2.57 ± 0.49
Mean % body ± S.D. (g)	1.05	0.96	0.97	1.09
Kidneys				
Mean weight ± S.D. (g)	2.36 ± 0.24	2.63 ± 0.13	2.27 ± 0.11	2.57 ± 0.16
Mean % body ± S.D. (g)	1.00	1.13	0.98	1.09

Notes: CG—control group; EG—experimental group.

**Table 7:** Biochemical data for blood samples of animals vaccinated with QazCoVac-P, a subunit COVID-19 vaccine (*n* = 10, × ± S.D.).

Groups	ALT(U/L)	AST(U/L)	TP (g/L)	T-BIL (μmol/L)	D-BIL (μmol/L)	ALP (U/L)	UREA (mMol/L)	CREA	GLU
CG #1	99.06 ± 22.3	84.42 ± 58.38	63.2 ± 2.44	7.48 ± 1.26	0.78 ± 0.03	516.3 ± 88.36	4.62 ± 1.57	64.76 ± 12.83	7.06 ± 0.79
EG #2	71.2 ± 4.3	153.28 ± 18.41	57.54 ± 2.52	6.78 ± 1.24	0.64 ± 1.10	576.72 ± 68.05	5.26 ± 0.71	51.9 ± 2.64	7.18 ± 0.17
EG #3	70.1 ± 11.2	187.52 ± 30.50	55.5 ± 3.0	6.1 ± 0.36	0.54 ± 0.04	579.4 ± 81.24	4.84 ± 1.44	57.16 ± 4.99	6.86 ± 0.43
EG #4	76.76 ± 14.2	139.82 ± 24.77	63.4 ± 9.12	7.2 ± 1.84	0.72 ± 0.22	513.04 ± 118.23	4.28 ± 1.57	68.88 ± 16.33	6.94 ± 0.63

Notes: CG—control group; EG—experimental group.

**Table 8:** Hematology data for blood samples of animals vaccinated with QazCoVac-P, a subunit COVID-19 vaccine (*n* = 10, × ± S.D.).

Groups	CG #1	EG #2	EG #3	EG #4
RBC (10^12^/L)	7.36 ± 2.08	6.10 ± 1.30	7.04 ± 0.24	8.06 ± 0.77
HGB (g/L)	138.25 ± 44.1	121.2 ± 24.9	142.8 ± 4.16	157.2 ± 9.52
HCT (%)	37.68 ± 10.84	29.79 ± 5.72	36.26 ± 1.15	43.31 ± 3.0
MCV (fL)	52.00 ± 1.3	51.4 ± 1.2	49.2 ± 1.3	54.00 ± 3.2
MCH (pg)	19.23 ± 0.49	20 ± 0.8	19.28 ± 0.4	19.6 ± 0.8
RDWc (%)	22.4 ± 6.6	15.78 ± 0.9	16.62 ± 0.8	21.56 ± 1.3
PLT (10^9^/L)	424.0 ± 92.0	539.0 ± 129.6	669.00 ± 77.6	589.4 ± 115.84
PCT (%)	0.33 ± 0.02	0.33 ± 0.07	0.43 ± 0.05	0.40 ± 0.08
MPV (fL)	6.7 ± 0.13	6.38 ± 0.3	6.88 ± 0.1	6.88 ± 0.1
WBC (10^9^/L)	5.97 ± 2.0	10.46 ± 5.4	12.00 ± 2.4	10.30 ± 2.7
LYM (10^9^/L)	50.5 ± 3.0	48.2 ± 2.16	45.6 ± 3.28	54.2 ± 4.16
MID (10^9^/L)	1.58 ± 0.4	2.62 ± 2.1	2.66 ± 0.8	2.17 ± 0.35
GRA (10^9^/L)	1.16 ± 0.2	2.43 ± 1.4	4.67 ± 2.6	2.20 ± 0.61
LYM (%)	50.5 ± 3.0	48.2 ± 2.16	45.6 ± 3.28	54.2 ± 4.16
MID (%)	13.29 ± 7.3	22.66 ± 8.7	23.24 ± 7.1	23.32 ± 6.7
GRA (%)	18 ± 4.6	26.04 ±9.2	36.16 ± 13.9	22.32 ± 7.6
Erythrocyte sedimentation rate (ESR)	1.00 ± 0.00	1.00 ± 0.00	1.00 ± 0.00	2.2 ± 1.92

Notes: CG—control group; EG—experimental group.

## Data Availability

Data is contained within the article.
